# Adolescents' offline meetings with online contacts: Longitudinal relationships between the behavior and perceptions of risks, benefits, and peer involvement

**DOI:** 10.1111/jora.70267

**Published:** 2026-09-22

**Authors:** Vojtěch Mýlek, Ainize Martínez‐Soto, Lenka Dedkova

**Affiliations:** ^1^ Interdisciplinary Research Team on Internet and Society, Faculty of Social Studies Masaryk University Brno Czechia

**Keywords:** adolescents, face‐to‐face meetings, longitudinal survey, online acquaintances, peer norms, risk–benefit perceptions

## Abstract

Adolescents commonly meet new people online, and some later meet them in person. Such face‐to‐face meetings offer benefits alongside risks, which are emphasized in public discourse. Thus, adolescents' perceptions of how risky, beneficial, and normative the meetings are key to understanding their decisions to meet. Yet research on their effects is scarce and mostly cross‐sectional. Drawing on the health belief model and the theory of planned behavior, this longitudinal study examines how perceived risk severity and likelihood, benefits likelihood, and peer involvement influence adolescents' face‐to‐face meetings with online contacts. We collected three waves of survey data from 2500 Czech adolescents (ages 11–16, 50.0% girls) and analyzed them using random intercept cross‐lagged panel models. Perceived risk severity predicted a decrease in the number of face‐to‐face meetings, while perceived benefits likelihood predicted an increase. Reciprocally, attending more meetings predicted higher benefits likelihood and lower perceived risk severity. These reciprocal effects suggest that adolescents consider the risks and benefits of face‐to‐face meetings and update their perceptions based on experience. Unexpectedly, an increase in perceived risk likelihood predicted more face‐to‐face meetings, and perceived peer involvement did not predict either risk–benefit perceptions or the behavior itself. We discuss the nuanced influence of perceptions on different forms of adolescents' online risk‐taking and implications for preventive efforts.

## INTRODUCTION

Adolescents show a heightened propensity for risk‐taking behaviors compared to both children and adults (Casey et al., 2011). Nowadays, many risk‐taking behaviors take place entirely online (e.g., sexting) or begin on the internet and continue offline. According to established frameworks such as the health belief model (Rosenstock, 1974) and the theory of planned behavior (Ajzen, 1991), perceptions of how risky, beneficial, or normative the behavior is guide behavioral intentions and actions. Understanding how adolescents negotiate these perceptions is particularly crucial for risk‐taking behaviors, as they represent modifiable cognitive factors that directly influence decision‐making. Despite their theoretical importance, research examining how adolescents perceive the risks, benefits, and peer norms regarding online‐initiated behaviors, and the extent to which these perceptions impact their actions, remains limited.

In this study, we specifically focus on one risk‐taking behavior—offline face‐to‐face meetings between adolescents and their online contacts, that is, the people they met online and know exclusively through the internet. Such meetings are relatively common among European adolescents and can be both risky and beneficial (Smahel et al., 2020). While most of these meetings are reported to be positive (Mýlek, Dedkova, & Mesch, 2023), they are also framed within a risk‐focused discourse that emphasizes threats such as sexual assault, stalking, or physical harm (Filice et al., 2022; Paat & Markham, 2021). Thus, investigating the role of adolescents' own evaluations of the potential risks and benefits in their decision to attend these meetings is particularly relevant. Further, since peers play a crucial role in adolescent decision‐making (Defoe et al., 2022) and perceptions about peer involvement predict risky online behaviors beyond evaluations of risks and benefits (Baumgartner et al., 2010), we also examine how perceived norms about peers influence adolescents' behavior. Nevertheless, previous research has mostly focused on other predictors, such as sensation‐seeking traits or online activity patterns (Mýlek et al., 2020; Van den Heuvel et al., 2012), and the role of adolescents' perceptions received little attention (e.g., Mýlek, Dedkova, & Smahel, 2023). At the same time, this research was cross‐sectional, limiting our ability to determine whether perceptions predict behavior or are shaped by it. Therefore, in this three‐wave longitudinal study, we investigate whether adolescents' perceptions of lower risks, greater benefits, or higher peer involvement predict later engagement in face‐to‐face meetings, and whether adolescents' own experiences of meeting online contacts subsequently influence their perceptions.

### Face‐to‐face meetings with online contacts

For youth growing up in digital societies, the Internet serves not only as a platform for communication and entertainment, but also as a space for maintaining social relationships and establishing new ones. Social media, online games, dating apps, and other digital platforms allow adolescents to connect with a broad range of people, and some of these connections transition from virtual to real‐world interactions (Smahel et al., 2020; Zhang et al., 2017). Meeting face‐to‐face with someone known only from the internet is relatively common for adolescents (11–16 years old), with reported yearly prevalence across Europe between 5% in France and 25% in Serbia (Smahel et al., 2020), and lifetime prevalence of 32% in Czechia (Mýlek, Dedkova, & Mesch, 2023).

Face‐to‐face meetings with online contacts entail both benefits and risks. Regarding the benefits, the co‐construction model posits that online spaces can serve as a playground for addressing developmental tasks related to identity exploration, peer affiliation, and intimacy (Subrahmanyam & Šmahel, 2011). Meeting online contacts offline provides an avenue for addressing these tasks by offering opportunities to make new friends, receive social support, or find a romantic partner. This is supported by evidence showing that most adolescents go to these meetings intending to encounter new people and make friends; they meet predominantly with peers of the same age and often stay in touch after the first meeting (Mýlek et al., 2024). Adolescents also mostly report that such meetings are positive experiences. On average across 15 European countries, 70% of adolescents felt happy after such meetings (Smahel et al., 2020), and in Czechia, 69% reported their last meeting had been pleasant (Mýlek et al., 2024).

However, face‐to‐face meetings also carry considerable risks, including meeting someone unexpected, sexual assault, or other victimization. Notably, only 9% of adolescents who experienced such a meeting across Europe felt upset after (Smahel et al., 2020), and 8% of Czech adolescents found their last meeting unpleasant (Mýlek et al., 2024). Yet, in a retrospective study of US undergraduates, 68% of those who recalled meeting an adult “online stranger” in person as minors (i.e., 6% of the whole sample) reported having sexual intercourse with the adult. While most of these experiences were anticipated and consensual, some were not (Greene‐Colozzi et al., 2020). These findings illustrate that meeting online contacts offline can include grave risks, which, though infrequent, should not be understated. It is worth noting that adolescents are aware of such risks (Mýlek, Dedkova, & Smahel, 2023) and actively employ safety strategies, for instance, to assess the age of their online contact (Groenestein et al., 2018).

Since these meetings offer developmental benefits alongside risks, they can be framed as instances of positive risk‐taking. This concept highlights that engaging in risky behavior is not always harmful, and that some degree of calculated risk‐taking can be developmentally adaptive (Crone & Dahl, 2012; Romer et al., 2017). In case of face‐to‐face meetings, such risk–benefit calculations occur within a polarized social context. On the one hand, the risks of meeting “online strangers” are amplified and often exaggerated in media narratives and public discourse (Korkmazer et al., 2020; Mascheroni et al., 2014). Qualitative research suggests that these risk‐focused narratives are reflected in adolescents' discourse (Mascheroni et al., 2014), and quantitative evidence shows that adolescents do perceive face‐to‐face meetings with online contacts as risky, though these perceptions vary by age and gender (Mýlek, Dedkova, & Smahel, 2023). At the same time, given the relatively high prevalence of these meetings, especially positive ones (Mýlek et al., 2024), adolescents are likely to know peers who have had positive experiences with them. Because adolescents are caught between these exaggerated public risk messages and tangible social rewards, their decisions to meet are likely driven by how they subjectively evaluate these competing factors.

Yet, previous research on predictors of such face‐to‐face meetings has predominantly focused on personal predispositions (e.g., sensation seeking), relational predispositions (e.g., family and peer relationships), or behavioral factors (e.g., time spent online, exposure to online risks) (e.g., Mýlek et al., 2020; Van den Heuvel et al., 2012). Although this research helped identify which adolescents are more likely to experience face‐to‐face meetings, less is known about the cognitions that drive adolescents' decisions to meet their online contacts. Since these meetings involve a mix of potential benefits and risks, and peer influence plays an important role in adolescents' risk‐taking and risk evaluations (Defoe et al., 2022; Knoll et al., 2015), we argue that adolescents' perceptions about risks, benefits, and peer involvement in face‐to‐face meetings are particularly relevant in this regard. These perceptions offer a distinct explanatory lens that go beyond the perspectives of personal traits. Unlike, for instance, sensation seeking (a dispositional tendency to seek novelty and intensity), which reflects a general trait‐level orientation, adolescents' perceptions are context‐specific cognitive evaluations that are tied to a particular behavior, malleable over time, and directly amenable to intervention. Evidence also suggests that perceptions of risky behavior predict behavior even when traits like sensation seeking are controlled for (Zimmermann, 2010). Thus, adolescents with similar individual dispositions may differ in their perceptions of a specific behavior, depending on their prior experiences, social environment, and available information; it is precisely these perceptions that we examine here.

### Risk‐taking and decision‐making in adolescence

Adolescence is a developmental period marked by heightened exploration, autonomy seeking, and sensitivity to social influence. This period is characterized by the increased tendency to engage in risk‐taking behaviors, from experimenting with substances to unsafe sexual activity or reckless driving (Blankenstein et al., 2024; Habib et al., 2023), which is partially driven by neural development. The brain's reward system undergoes rapid development, whereas impulse control and long‐term planning mature more gradually (Casey et al., 2011). This imbalance contributes to heightened impulsivity, sensation seeking, and prioritization of immediate rewards over distant costs. As a result, adolescents are more likely to be reward‐oriented and to engage in novel or high‐arousal activities, often without fully assessing long‐term consequences (Collins et al., 2012; Joseph et al., 2009). Moreover, when evaluating risks, adolescents are more susceptible to social influence than children or adults (Knoll et al., 2015), a pattern that may be linked to their brain development (Knoll et al., 2020).

Despite the neurodevelopmental underpinnings of risk‐taking, adolescents are not incapable of recognizing risks, and their conscious decision‐making plays a pivotal role. Adolescents can refrain from risky behavior when inhibitory control is engaged, but doing so requires greater effort compared to adults (Reyna & Farley, 2006). In fact, according to the fuzzy trace theory, adults tend to rely on heuristic representations that some behaviors are inherently dangerous and should be avoided (i.e., *gist representations*), while adolescents rely more on *verbatim representations*, which involve weighing the potential costs and benefits of the risky behavior (Reyna & Farley, 2006; White et al., 2018), making the assessment on both risk and benefits crucial. Two prominent theoretical frameworks offer insight into these evaluative processes: the health belief model (Rosenstock, 1974) and the theory of planned behavior (Ajzen, 1991). Both frameworks share a fundamental logic: perceived risks deter behavior while perceived benefits promote it.^1^

The health belief model (Rosenstock, 1974) proposes three types of risk–benefit perceptions, which we also adopt here: perceived susceptibility (i.e., the likelihood that a negative outcome will occur), perceived severity (i.e., the magnitude of harm if it does occur), and perceived benefits. This distinction is important as adolescents may recognize that face‐to‐face meetings with online strangers carry potentially serious consequences (high severity, e.g., sexual assault) while seeing them as unlikely to happen to them personally (low susceptibility). Conversely, they may perceive certain risks as highly probable (high susceptibility, e.g., awkward meeting) but relatively inconsequential (low severity). Both dimensions may independently influence decision‐making, with severity likely playing a particularly salient role when serious harm may occur (Orji et al., 2012). Additionally, because adolescents tend to be more reward‐oriented, perceived benefits may take precedence over perceived risks when they make decisions about potentially risky behaviors.

The theory of planned behavior (Ajzen, 1991) posits that behavior is guided by attitudes toward the behavior, subjective norms (i.e., beliefs about important others' approval of the behavior), and perceived behavioral control (i.e., beliefs about one's ability to perform the behavior). While risk and benefit perceptions are not included explicitly, they can be conceptualized as behavioral beliefs (i.e., expectations about positive or negative behavior outcomes) that contribute to the positive or negative attitudes toward behavior (Ajzen, 2020), thus making the respective behavior more or less likely to occur. Notably, through the construct of subjective norms, that is, individuals' perceptions of whether important others approve or disapprove of a behavior (Ajzen, 1991), this theory also emphasizes the role of social influence on behavior. Social norms theory offers further refinement by distinguishing between descriptive norms (i.e., beliefs about how common a behavior is among peers) and injunctive norms (i.e., beliefs about peers' approval of the behavior) (Larimer et al., 2004). Both frameworks recognize that adolescents who believe their friends engage in or approve of specific behaviors are more likely to adopt those behaviors themselves—a notion that is consistently supported by research on offline risky behaviors (e.g., smoking, substance use, risky sex behaviors) (Defoe et al., 2022).

In summary, according to the health belief model and theory of planned behavior, adolescents who perceive lower susceptibility to harm, view potential negative outcomes as less severe, and anticipate greater benefits should be more likely to engage in face‐to‐face meetings with online contacts. Additionally, based on the theory of planned behavior and social norms theory, adolescents who believe more of their peers engage in these meetings should be more likely to engage in them themselves.

### Adolescent perceptions and online risk‐taking

Adolescents' decision‐making processes about risk‐taking behaviors are particularly relevant in the digital age, where online interactions create new opportunities for risky behavior. Although empirical evidence supports (some of) these theoretical links outlined above, the research on adolescents' cognitions in this context is relatively limited. Mýlek, Dedkova, and Smahel (2023) found that Czech adolescents who view the risks of face‐to‐face meetings with people met online as more severe are less inclined to participate in such meetings. Similarly, perceiving online contact with strangers as risky was associated with reduced engagement in these contacts (Dedkova & Mýlek, 2023). Research on other forms of online risky behavior provides additional evidence. A longitudinal study found that Dutch adolescents who perceived online sexual activities as risky or themselves as vulnerable to such risk were less likely to engage in such activities 6 months later (Baumgartner et al., 2010). Additionally, Malaysian adolescents who rated online risks as more severe were less likely to participate in behaviors such as sexting or seeking intimate contacts online (Teimouri et al., 2018). Together, these findings suggest that risk perceptions are meaningfully related to adolescent online risk‐taking. However, only one of these studies examined benefit perceptions, finding no effect on adolescents' online risky sexual behaviors (Baumgartner et al., 2010). Moreover, this study was also the only one using a longitudinal design. Thus, there are important gaps in understanding the decision‐making process—especially regarding the role of benefit perceptions and potential reciprocal effects.

The dynamics of peer influence may differ between offline and online risk contexts. Offline risk behaviors (e.g., smoking, reckless driving, drinking) often occur in social settings where peers are physically present, creating direct peer pressure and immediate social rewards for participation. In contrast, face‐to‐face meetings with people met online are typically more private decisions, made and carried out without peers present. This reduced visibility may diminish direct peer pressure, though it need not eliminate social influence entirely. Adolescents may still form perceptions about whether their friends engage in or approve of such meetings, even in the absence of open discussion or observable behavior. A longitudinal study found that Dutch adolescents who perceived their friends as engaging in online sexual activities were more likely to engage in such behaviors themselves 6 months later, even after controlling for risk and benefit perceptions (Baumgartner et al., 2010). This demonstrates that peer norms effects exist independently of individual risk assessments. Another longitudinal study showed that descriptive norms (i.e., perceived peer involvement) consistently predicted adolescents' own engagement in risky online sexual behavior. Injunctive norms (i.e., perceived peer approval) showed weaker and less consistent effects (Baumgartner et al., 2011). This suggests that in online contexts, where behaviors are less publicly visible, beliefs about peer involvement are more influential than beliefs about peer approval. Since previous studies have not examined these effects in face‐to‐face meetings, we aim to fill this gap by focusing on the role of perceived peer involvement.

Apart from directly influencing behavior by setting a norm, perceived peer involvement may also shape adolescents' risk and benefit perceptions. Although the theory of planned behavior typically treats subjective norms and attitudes as parallel predictors of behavioral intentions (Ajzen, 1991), there are reasons to expect that social information influences evaluations of risks and benefits. Knoll et al. (2015) found that peers' evaluations of risk influenced adolescents' risk perceptions in various hypothetical scenarios, suggesting that social context shapes cognitive evaluations of danger. Furthermore, learning about face‐to‐face meetings from peers who had already attended such meetings was associated with lower risk perception among girls (Mýlek, Dedkova, & Smahel, 2023), indicating that social sources of information may attenuate perceived risk. When adolescents observe or believe that many of their peers attend face‐to‐face meetings with online contacts, they may interpret it as evidence that such meetings are not particularly dangerous or that they offer meaningful benefits. Thus, peer norms may function not only as direct motivators but also as informational cues that calibrate individual risk–benefit assessments.

### Reciprocal effects of face‐to‐face meetings on perceptions

While adolescents' perceptions can predict risky behavior, participation in such behaviors can also reshape perceptions. Drawing from the cognitive dissonance theory (Festinger, 1957), adolescents who have engaged in risky acts may minimize perceived harm and highlight potential benefits to justify their choices (Gerrard et al., 1996). The theory of planned behavior similarly acknowledges feedback effects, recognizing that engaging in a behavior can alter attitudes toward that behavior (Ajzen, 2020). Crucially, the available evidence suggests that face‐to‐face meetings with people met online are predominantly positive experiences. Across Europe, 70% of adolescents reported feeling happy after meeting and online contact in person (Smahel et al., 2020), and in Czechia, 69% described their last such meeting as pleasant (Mýlek et al., 2024). Adolescents who attend more meetings are therefore likely to accumulate mostly positive experiences. This would naturally attenuate perceived risks, as the feared negative outcomes repeatedly fail to materialize, and simultaneously reinforce perceived benefits, as the social rewards of meeting become more concrete and tangible. In this way, attending more meetings is expected to affect both risk and benefit perception: perceived risk declines because exposure disconfirms anticipated danger, while perceived benefit strengthens because experience confirms the positive value of such meetings. Adolescents who attend face‐to‐face meetings and have a negative experience, by contrast, might heighten risk perceptions. Thus, longitudinal studies are necessary to clarify whether perceptions predict risky behavior or form as consequences of such behavior. Baumgartner et al.'s (2010) longitudinal study found that risk perceptions predicted online sexual behavior, but the reverse effect, behavior predicting perception, was not detected. To date, no longitudinal study has investigated similar effects for face‐to‐face meetings with people met online, leaving the directionality and predictive utility of risk–benefit perceptions unclear.

### The present study

The current study seeks to advance understanding of adolescents' engagement in online‐initiated risk‐taking, specifically, face‐to‐face meetings with people first met online. Previous research has shown that adolescents' perceptions of risks, benefits, and peer involvement play a central role in decision‐making about offline risk behaviors, yet little is known about how these perceptions relate to meeting online contacts face‐to‐face. Moreover, most existing studies have relied on cross‐sectional designs, leaving unclear whether perceptions of risk and benefit predict engagement in these meetings or are influenced by it. Thus, the main goal of this three‐wave longitudinal study is to examine how adolescents' perceived risks (likelihood and severity), perceived benefits, and perceived peer involvement (i.e., descriptive peer norms) are linked to their face‐to‐face meetings with people first met online. We leverage the longitudinal design to separate between‐person differences from within‐person processes. This allows us to control for stable individual differences and focus on how intra‐individual changes in perceptions drive changes in behavior (and vice versa) over time (Hamaker et al., 2015). Our hypotheses focus on within‐person effects, specifically, how deviations from an individual's trait‐level mean at one wave predict subsequent within‐person deviations 6 months later:

Adolescents' perceived risk likelihood (a) and severity (b) negatively predict the number of attended face‐to‐face meetings, whereas perceived benefit likelihood (c) positively predicts the number of such meetings.

The number of attended face‐to‐face meetings negatively predicts adolescents' perceived risk likelihood (a) and severity (b) and positively predicts perceived benefit likelihood (c).

Perceived peer involvement positively predicts the number of face‐to‐face meetings.

Perceived peer involvement negatively predicts adolescents' perceived risk likelihood (a) and severity (b) and positively predicts perceived benefit likelihood (c).

## METHODS

### Sample and procedure

We used data from a multifocal longitudinal online survey of Czech parents and adolescents. The data were collected in three waves spaced 6 months apart, between June 2021 and June 2022. A survey agency DataCollect sampled panelists from three large online panels (i.e., ivyzkumy.cz, MNforce ePanel, Kantar; together approx. 165,000 panelists) and 980 newly recruited households. We set quotas for household SES (head of household education), municipality population, and region (14 regions based on NUTS3; European Commission & Eurostat, 2020); all respective to Czech households with children. Additionally, quotas for adolescent age and gender ensured equally sized and gender‐balanced cohorts. Sample description is available in our Open Science Framework (OSF) repository at https://osf.io/psrfa.

Prior to data collection, we tested our questionnaire in 30 semi‐structured cognitive interviews and a quantitative pilot on 195 adolescent–parent dyads. For the main data collection, the agency contacted panelists (i.e., parents) via email, providing information about the study and a link to the questionnaire. Both the participating parents and adolescents consented to their participation. The parents could review the adolescent questionnaire before consenting. Parents and adolescents were instructed to fill in the questionnaire independently and privately. After each wave, each household received a monetary reward according to the agency's standards. The data collection was approved by the Research Ethics Committee of Masaryk University.

In T1, we collected data from 2500 adolescents (ages 11–16, *M* = 13.43, *SD* = 1.70, 50.0% girls), which reduced to 1654 in T2 (33.8% attrition) and 1102 in T3 (33.4% attrition). We used logistic regression to test whether attrition was related to studied variables. None of these variables, that is, adolescents' age and gender, number of face‐to‐face meetings, perceived peer involvement, and risk and benefit perceptions, significantly predicted whether adolescents responded in all waves (*n* = 1102) or dropped out at any point (*n* = 1398; *χ* ^2^(7) = 5.75, *p* = .569).

### Measures

All items, apart from those asking about age and gender, included a response option “I don't want to respond,” treated as a missing value. See Table 1 for descriptive statistics, scale reliability, and T1 correlations. Measures are available via OSF at https://osf.io/psrfa.

**TABLE 1:** Descriptive statistics and T1 Pearson correlations.

Variable	Range	T1 (*N* = 2500)			T2 (*N* = 1654)			T3 (*N* = 1102)			Pearson correlations (T1)					
		*M* (*SD*)	Miss %	*ω*	*M* (*SD*)	Miss %	*ω*	*M* (*SD*)	Miss %	*ω*	G	A	BL	RL	RS	PI
Age (A)	11–16	13.43 (1.70)	0.0								.02					
Benefits likelihood (BL)	1–4	2.23 (0.72)	2.4	.87	2.16 (0.70)	0.6	.87	2.13 (0.72)	0.5	.89	.01	.16***				
Risks likelihood (RL)	1–4	2.32 (0.87)	9.9	.86	2.43 (0.88)	3.4	.89	2.42 (0.87)	3.2	.88	−.05*	−.06**	−.13***			
Risks severity (RS)	1–4	3.35 (0.73)	2.6	.86	3.33 (0.72)	1.5	.87	3.30 (0.74)	0.9	.89	−.09***	−.05*	−.14***	.26***		
Peer involvement (PI)	1–5	2.32 (1.09)	2.4		2.26 (1.04)	1.0		2.30 (1.04)	1.2		.03	.17***	.37***	−.13***	−.20***	
Face‐to‐face meetings	1–5	1.24 (0.81)	3.7		1.38 (0.84)	1.3		1.36 (0.84)	1.3		−.01	.11***	.21***	−.14***	−.07***	.25***
1 *none*		89.5%			78.4%			79.6%								
2 *one*		3.6%			11.0%			10.8%								
3 *two*		2.8%			6.4%			5.4%								
4 *three*		1.3%			2.6%			2.5%								
5 *four or more*		2.7%			1.5%			1.7%								

*Note*: G = Gender (1 = *girl*, 2 = *boy*). Miss % = percent of missing values discounting attrition. Mean scores for benefits likelihood, risks likelihood, and risks severity.

* *p* < .05.

** *p* < .01.

*** *p* < .001.

#### Perceived peer involvement

To assess adolescents' perceptions about peer involvement in face‐to‐face meetings with online acquaintances, we first defined online acquaintances (“On the internet, people can have conversations with other people whom they do not know from real life ‐ they have not met in person. These conversations can happen in various places, e.g., on social networks, in games, on dating sites, in internet discussion, etc. We are not talking about” professional “communication (e.g., with e‐shop, tutor, helpline)”). Then, we asked, “Some people also meet people they only from the internet face to face – in reality. In your opinion, how common or uncommon is it to go to such meetings among your friends?” providing five response options (1 = *very uncommon*—5 = *very common*).

#### Face‐to‐face meetings

We measured the number of face‐to‐face meetings with online acquaintances that adolescents experienced with one item: “How many such meetings have you experienced in [T1: your life; T2, T3: in the past 6 months]? Here we do not mean repeated meetings with the same person, but only those meetings where you met someone new” (1 = *none*—5 = *four or more*). In T1, the item asked about a broader period (in your life”). To account for this discrepancy, we used information from a follow‐up item that asked how long ago the most recent meeting took place. We recoded the T1 responses of adolescents who reported this meeting happened more than 6 months ago to 1 = *none*. This way ensured the same timeframe for the item in all waves.

#### Risk and benefit perceptions

We measured perceptions about the risks and benefits of face‐to‐face meetings using seven newly developed items. Four items described potential benefits (e.g., “I would meet a new friend or partner.”) and three the potential risks (e.g., “The person who would come to the meeting would be different from what I expected.”). To assess *perceived risks/benefits likelihood*, adolescents were instructed to imagine they are about to go to a face‐to‐face meeting and estimate how likely these scenarios are to happen (1 = *not at all likely*—4 = *very much likely*). To assess *perceived risks severity*, we used the same three items with potential risks, asking adolescents how dangerous it would be were they to happen (1 = *not at all dangerous*—4 = *very much dangerous*).

A three‐factor CFA model fit the T1 data well, *χ* ^2^(32) = 359.28, *p* < .001, CFI = 0.97, TLI = 0.95, RMSEA = 0.07 with 90% CIs [0.06; 0.08], SRMR = 0.04, and all three subscales exhibited good reliability in each wave (see Table 1). In testing for longitudinal invariance, the configural model fit well, *χ* ^2^(339) = 1017.47, *p* < .001, CFI = 0.97, TLI = 0.96, RMSEA = 0.03 with 90% CIs [0.03; 0.03], SRMR = 0.04, and the metric model did not fit significantly worse, *χ* ^2^(14) = 21.52, *p* = .089. While the scalar model fit significantly worse that the metric model, *χ* ^2^(14) = 28.39, *p* = .013, the difference in fit was negligible, ΔCFI < 0.001, ΔRMSEA < 0.001, ΔSRMR < 0.001. Thus, the scale was scalar invariant across time.

#### Age and gender

We asked about adolescents' age and binary gender (“Are you…”, 1 = *a girl*, 2 = *a boy*).

### Analysis

We tested our hypotheses using a random intercept cross‐lagged panel model (RI‐CLPM; Hamaker et al., 2015). The RI‐CLPM expands on the cross‐lagged panel model by decomposing variables into random intercepts, which capture individuals' typical levels, stable across time, and within‐person centered variables, which capture individuals' deviations from these person‐specific typical levels at each time point. The resulting model has two parts. The within‐person part includes *cross‐lagged effects*, which capture how deviations from the typical level in one variable predict such deviations in another variable in the next time point, and *autoregressive effects*, which capture how such deviations predict deviations in the same variable in the next time point, that is, the extent to which such deviations carry over in time. The between‐person part includes covariances between random intercepts, that is, the stable, trait‐like differences between people, and the effects of any time‐invariant predictors (see Extension 1; Mulder & Hamaker, 2021).

We estimated a RI‐CLPM including five time‐varying variables (i.e., face‐to‐face meetings, peer involvement, benefits likelihood, risks likelihood, and risks severity). We also included adolescents' age and gender as time‐invariant predictors to control for their effects at the between‐person level (see Extension 1; Mulder & Hamaker, 2021). We modeled face‐to‐face meetings and peer involvement as observed variables, and risk/benefit perceptions as latent factors (see Extension 3; Mulder & Hamaker, 2021). We simplified the model by omitting cross‐lagged effects between the three risk/benefits perception variables. We estimated two variations of this model as robustness checks: one without the time‐invariant predictors, and another that included cross‐lagged effects between risk and benefit perceptions. All models were estimated using maximum likelihood estimation with robust standard errors (MLR) and handled missing values using full information maximum likelihood (FIML). We conducted modeling in Mplus v8.11 and additional analyses in R. Data, analytical scripts, and results of all analyses are available via OSF at https://osf.io/psrfa.

## RESULTS

**FIGURE 1:**
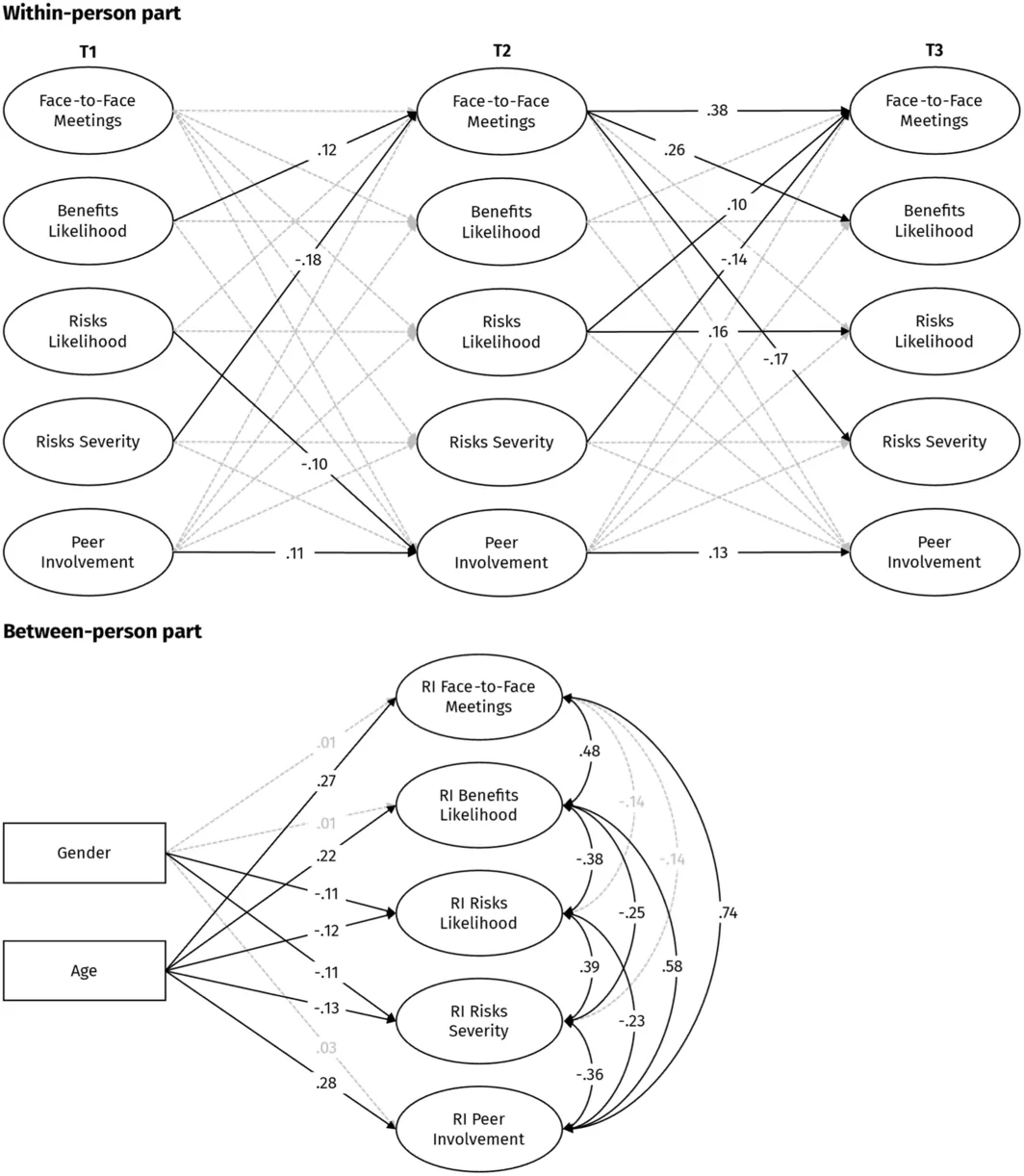
Simplified results of the RI‐CLPM (*N* = 2500). RI, random intercept. Standardized results (STDYX). Although presented across two diagrams, the results reflect a single RI‐CLPM model. Omitted for clarity: Latent variables' indicators, covariances between within‐person centered variables and their residuals, decomposition into random intercepts and within‐person centered variables.

Our RI‐CLPM model fit the data well, *χ* ^2^(593) = 1801.08, *p* < .001, CFI = 0.96, TLI = 0.95, RMSEA = 0.03 with 90% CIs [0.03; 0.03], SRMR = 0.04. See Figure 1 for simplified results overview, Appendix A for more detailed results, and OSF repository for full results of all models.

### Within‐person effects

We found one significant cross‐lagged effect that was consistent across the three waves. Deviations in perceived risks severity negatively predicted deviations in the reported number of face‐to‐face meetings 6 months later, supporting H1b. Other significant cross‐lagged effects were significant only from T1 to T2 or from T2 to T3. Perceived risks likelihood negatively predicted perceived peer involvement (T1 → T2; not hypothesized), and positively predicted face‐to‐face meetings (T2 → T3), providing evidence of an opposite effect than expected in H1a. Perceived benefits likelihood positively predicted face‐to‐face meetings (T1 → T2), partially supporting H1c. Face‐to‐face meeting positively predicted perceived benefits likelihood and negatively predicted perceived risks severity (both T2 → T3), partially supporting H2b and H2c. All other cross‐lagged effects were not significant, that is, H2a, H3, H4a–c were not supported. Regarding autoregressive effects, the autoregressive effect for peer involvement was significant in all waves (i.e., T1 → T2, T2 → T3), for face‐to‐face meetings and perceived likelihood or risks only T2 → T3, and there were no significant autoregressive effects for perceived benefits likelihood and risks severity.

### Between‐person associations

On the between‐person level, adolescents who reported more face‐to‐face meetings also perceived the benefits of such meetings to be more likely and reported the meetings are more common among their peers. Perceived benefits likelihood was positively associated with higher perceived peer involvement and lower perceived risks likelihood and severity. Perceived risks likelihood was positively associated with perceived risk severity, and both risk perceptions were negatively associated with perceived peer involvement.

Regarding time‐invariant predictors, older adolescents reported engaging in more face‐to‐face meetings, higher perceived benefits likelihood, and higher perceived peer involvement. In contrast, older adolescents reported lower perceived risks likelihood and severity. Adolescent boys also reported lower perceived risks likelihood and severity. However, adolescents' gender was unrelated to perceived benefits likelihood, perceived peer involvement, and the number of face‐to‐face meetings attended.

### Robustness checks

We tested the robustness of our findings by comparing the pattern of reported results to the results of two variations of our model. First, removing age and gender as time‐invariant predictors did not lead to any notable changes. Second, adding the 12 cross‐lagged paths between benefits likelihood, risks likelihood, and risks severity produced one additional cross‐lagged effect: T1 benefits likelihood positively predicted T2 perceived peer involvement. Otherwise, the results remained unchanged. This suggests that our results are robust toward minor changes in model specification.

## DISCUSSION

This longitudinal study examined whether adolescents' perceptions related to the number of meetings with online acquaintances face‐to‐face influence their behavior, that is, a specific risky behavior at the intersection of offline and online environments. Our findings support bidirectional effects for perceptions of risks and benefits, and limited effects for perceived peer involvement. Changes in adolescents' risk and benefit perceptions, especially the perceived severity of risks, predicted changes in the number of their face‐to‐face meetings that, in turn, predicted changes in risk and benefit perceptions (albeit less consistently over time). The findings partially align with decision‐making theories, such as the health belief model (Rosenstock, 1974) or the theory of planned behavior (Ajzen, 1991), while also revealing important nuances about what specific perceptions matter and which risk‐taking behaviors they apply to.

### Reciprocal effects of risk and benefit perceptions

Our analysis provided the most robust evidence for the effect of perceived severity of risks. The negative within‐person effect suggests that an increase in adolescents' perception of how dangerous the potential risks of face‐to‐face meetings are (e.g., meeting someone unexpected, being forced to do something unwanted), predicted a decrease in the number of face‐to‐face meetings adolescents reported 6 months later. This effect showed consistently across waves, supporting Hypothesis 1b. It corroborates previous cross‐sectional studies, which linked higher risk perception to less online contact with people met online (Dedkova & Mýlek, 2023) and lower likelihood of meeting such people face‐to‐face (Mýlek, Dedkova, & Smahel, 2023). It is also in line with a longitudinal study, where adolescents who perceived risky online sexual behaviors as more dangerous participated in such behaviors less in the following 6 months (Baumgartner et al., 2010). Together, these findings support the core ideas of decision‐making theories, such as the health belief model or the theory of planned behavior, and show that perceiving higher risks detracts adolescents from engaging in risk‐taking behaviors, including meeting face‐to‐face with people known exclusively from the internet.

However, within‐person deviations in perceived likelihood of risks (sometimes labeled as risk vulnerability or susceptibility) did not predict meetings from T1 to T2 and had an opposite effect than we expected in Hypothesis 1a from T2 to T3. Between these waves, when adolescents' perception of how likely a risky situation is to happen to them increased, their later participation in face‐to‐face meetings with people from the internet also increased. This finding runs contrary to the aforementioned longitudinal study on risky online sexual behaviors, where the perceived vulnerability predicted such behaviors negatively (Baumgartner et al., 2010). This surprising positive effect might reflect that adolescents start realizing the potential risks once they begin to plan face‐to‐face meetings. Most adolescents do not meet their online acquaintances spontaneously and about 41% met them after at least 1 month of online contact (Mýlek et al., 2024). Thus, adolescents who reported face‐to‐face meetings between T2 and T3 could have started planning them already prior to T2. While planning, these adolescents may have considered the associated risks more concretely or sought additional information. Since the media discourse emphasizes the risks of meeting people from the internet (Dedkova, 2015; Holmes, 2009), seeking information about such meetings likely increased the perceived probability of risks. In other words, adolescents' intentions to meet may have increased both the likelihood of meetings in the next 6 months and the perceived likelihood of risks, explaining the unexpected positive effect. This interpretation also explains the absence of the effect from T1 to T2. We collected T1 data when contact restrictions related to the COVID‐19 pandemic were ending in Czechia (Slabá, 2022). Thus, prior to T1, potentially fewer adolescents were making plans for face‐to‐face meetings between T1 and T2.

This pattern may also reflect adolescents' broader developmental orientation toward risk‐taking. As outlined earlier, adolescents' decision‐making relies more on weighing concrete costs and benefits than on the categorical avoidance heuristics that guide adult risk‐avoidance (Reyna & Farley, 2006). Rather than deterring behavior, a rising awareness of risk likelihood may simply reflect adolescents' engagement in the reasoned evaluation process that precedes action; consistent with their heightened sensitivity to reward and novelty (Casey et al., 2011; Collins et al., 2012), they may proceed with planned meetings even while acknowledging elevated risk, provided the anticipated benefits and manageable severity outweigh the perceived likelihood of harm. Nevertheless, further research that would consider adolescents' intentions (and their timing) is necessary to test this interpretation.

Our evidence for the effect of perceived likelihood of benefits aligns with decision‐making theories. The positive within‐person effect implies that an increase in adolescents' perception of how likely they are to benefit from face‐to‐face meetings (e.g., make new friends, have fun, receive support) predicted an increase in the number of these meetings. Yet, we detected this effect only from T1 to T2, providing partial support to Hypothesis 1c. The timing of this effect may be tied to the pandemic context of our study. T1 data collection coincided with the easing of COVID‐19 contact restrictions in Czechia (Slabá, 2022), following a prolonged period of isolation. Adolescents emerging from this period may have anticipated unusually high benefits from face‐to‐face meetings with online contacts, prioritizing the novelty, social connection, and relief from isolation. This could have amplified the link between perceived benefits and subsequent meetings, specifically during the T1–T2 interval. It is thus possible that this specific context allowed capturing this theoretically assumed effect, which might be otherwise more difficult in observational design without external prompts affecting changes in benefits perceptions.

Notably, the effect of perceived benefits was not significant in the aforementioned longitudinal study on risky online sexual behaviors (Baumgartner et al., 2010). This inconsistency may reflect that Baumgartner et al. (2010) measured the magnitude of benefits, while we focused on their perceived likelihood. Alternatively, it may reflect that risky online sexual behavior and face‐to‐face meetings with online acquaintances represent different types of risk‐taking behaviors—*reactive* and *reasoned* (Maslowsky et al., 2019). Risky online sexual behavior, which includes looking for sexual partners online or sending nude pictures of oneself, may more often take the form of *reactive risk‐taking*, driven by momentary impulses (e.g., sexual arousal) and reactions to other people's behavior (e.g., responding to sexual solicitation during online flirting). Since face‐to‐face meetings usually require planning, they are more likely to be *reasoned risk‐taking*, driven more by the evaluation of potential risks and benefits. This suggests that different online risk‐taking is guided by different mechanisms, likely depending on how impulse‐driven and immediately achievable it is. Such differentiation bears implications for prevention—for more reasoned risk‐taking, a discussion of potential risks and benefits can be fruitful, while for more reactive risk‐taking behaviors, prevention should focus more on teaching adolescents to resist the momentary impulse for action and take time to consider the potential risks.

Apart from risk and benefit perceptions affecting behavior, our results also show the reverse effect. The within‐person effects suggest that when adolescents went to more face‐to‐face meetings at T2, they later (at T3) perceived the risks severity to be lower and the benefits likelihood to be higher, supporting Hypotheses 2b and 2c. The effects were only significant from T2 to T3, not from T1 to T2. A potential explanation is that due to COVID‐19‐related contact restrictions, fewer adolescents engaged in face‐to‐face meetings prior to T1 (see Table 1), which made the effects less pronounced from T1 to T2. Since most adolescents report their last face‐to‐face meeting was pleasant, and they felt happy after it (Mýlek, Dedkova, & Mesch, 2023; Smahel et al., 2020), the changes in perceived likelihood of benefits and severity of risks likely mean that adolescents updated these perceptions based on their experiences, in line with the Theory of Planned Behavior (Ajzen, 2020). Adolescents could also learn from their experiences, becoming more confident they can handle the risks or make the future meetings more enjoyable. Alternatively, going to a meeting despite considering it risky may trigger cognitive dissonance (Festinger, 1957). Thus, adolescents may adjust their perceptions about the associated risks and benefits to restore congruence between their perceptions and behavior. Notably, the previous longitudinal study of adolescents' risky online sexual behavior did not provide any evidence that involvement in such behaviors changes adolescents' risk and benefit perceptions 6 months later (Baumgartner et al., 2010). This discrepancy challenges the generalizability to other, potentially more reactive forms of online risk‐taking.

While going to more face‐to‐face meetings predicted a decrease in perceived severity of risks, it did not predict changes in their perceived likelihood (i.e., the within‐person effect was not significant), contrary to Hypothesis 2a. This asymmetry can be explained through the distinct nature of these perceptions and how they respond to experience. Risk likelihood reflects base rate information (i.e., what percentage of meetings “goes wrong”), which draws primarily on external, factual sources such as news reports, statistics, and public and educational warnings. In contrast, risk severity reflects personal impact assessment (i.e., “how bad it would be for me”), which is inherently subjective and experiential. When meetings go well, adolescents cannot “logically” conclude that risks never happen, but they can conclude that they personally possess the judgment and skills to prevent severe outcomes for themselves. This distinction aligns with psychological research differentiating *worry*, the experience‐resistant cognitive concern about future possibilities, from *fear*, the immediate emotional response to specific threats, which is highly responsive to direct experience (Borkovec et al., 1983). After positive meetings, adolescents feel less afraid (i.e., severity decreases) but remain cognitively aware of possibilities (i.e., likelihood remains stable).

Since the effects between adolescents' perceptions and face‐to‐face meetings are reciprocal, there is a possibility of a spiraling effect. Positive experiences can lead to more favorable risk–benefit perceptions, which, in turn, lead to more meetings. This pattern aligns with findings that face‐to‐face meetings become more common as adolescents age (Mýlek, Dedkova, & Mesch, 2023) and is consistent with the positive autoregressive effect we observed in our data.

### Perceived peer involvement

The within‐person effects of perceived peer involvement were not significant, implying that changes in perceived peer involvement in face‐to‐face meetings did not predict either adolescents' risk and benefit perceptions or their involvement in such meetings. Thus, Hypotheses H3 and H4a–c were not supported. This runs contrary to previous longitudinal studies, where perceived peer involvement positively predicted adolescents' risky online sexual behaviors 6 months later (Baumgartner et al., 2011), and the effect was larger than those of risk and benefit perceptions (Baumgartner et al., 2010). These contrasting results may reflect genuine behavioral differences. Descriptive norms (perceptions of what peers do) most influence behavior that is visibly socially performed, or subject to peer evaluation (Cialdini & Goldstein, 2004). Risky online sexual behavior may be more subject to peer influence as adolescents discuss such behaviors with peers in ways that create social validation, competition, or pressure (Widman et al., 2016). In contrast, face‐to‐face meetings with online strangers, while sometimes discussed, may be more privately decided and individually motivated. Because our measure captured perceived descriptive norms (i.e., perceptions of what peers do), rather than broader forms of peer influence, these findings should not be interpreted as evidence that peers are unimportant for adolescents' decisions. Instead, they suggest that adolescents' perceptions of how common these meetings are among their peers may be less influential for within‐person changes in meeting behavior than personal risk–benefit perceptions. Other forms of peer influence, such as peer approval, encouragement, or direct discussions about these meetings, may still shape adolescents' decisions but were beyond the scope of the present study.

Alternatively, the discrepancy can be ascribed to analytical differences. Our study employed RI‐CLPM, where the cross‐lagged paths capture within‐person effects and separate time‐stable differences between people. Since the previous longitudinal studies did not separate within‐ and between‐person effects, their estimates of cross‐lagged effects might be confounded by the stable between‐person differences (Hamaker et al., 2015). Indeed, while the cross‐lagged effects differed, T1 bivariate correlations between behavior and perceived peer involvement were positive both in previous studies (*r* = .43, .39; Baumgartner et al., 2010, 2011) and in this study (*r* = .25). Moreover, while perceived peer involvement did not predict face‐to‐face meetings on the within‐person level, it was closely correlated on the between‐person level. Therefore, as we discuss below, the between‐person association of perceived peer involvement with face‐to‐face meetings (and, potentially, with risky sexual online behavior in previous studies) is likely due to other factors and not because of causal relationships between the perception and the behavior.

An additional consideration concerns the nature of the perceived peer involvement measure itself. The measure assessed adolescents' perceptions of how common face‐to‐face meetings with online contacts are among their friends, rather than peers' actual behavior. Because these meetings are relatively private, adolescents may have limited direct knowledge of their peers' experiences and instead rely partly on inferences based on their own behavior and attitudes. This pattern is consistent with the false consensus effect (Ross et al., 1977), whereby individuals tend to overestimate how common their own behaviors are among others. If perceived peer involvement partly reflects adolescents' own behavior rather than an independent social cue, it would be expected to remain relatively stable and closely tied to their own general tendencies over time. Consequently, although the RI‐CLPM appropriately isolates within‐person processes, this characteristic of the measure may have reduced the likelihood of detecting within‐person peer effects across 6 months because changes in perceived peer involvement would provide little information beyond adolescents' own meeting behavior.

A further explanation concerns the perceived social acceptability of face‐to‐face meetings with online contacts. Media coverage often frames such meetings as dangerous, frequently associating them with “online predators” (Dedkova, 2015; Holmes, 2009). Given this pervasive narrative, adolescents may perceive such meetings as stigmatized behavior rather than an openly discussed and normalized peer activity. If face‐to‐face meetings with online contacts are not perceived as an accepted peer norm, descriptive‐norm mechanisms may simply not operate here the way they do for more openly discussed and performed risk behaviors. Future research should examine whether adolescents perceive these meetings as stigmatized and whether that perception moderates the influence of peer norms.

### Between‐person associations

On the between‐person level, our results show that adolescents who attend more face‐to‐face meetings with online contacts also perceive that the meetings are more common among their peers and their benefits are more likely. Notably, the associations with perceived likelihood and severity of risks were not significant. This pattern reveals that stable individual differences may be driven by approach rather than avoidance motivations. It suggests that individual differences in meeting attendance stem not from risk dismissal or ignorance, but from having genuine opportunities and reasons to meet. The quality and depth of adolescents' online relationships create systematic differences in both opportunities and perceptions. Adolescents who form deeper, meaningful connections characterized by greater self‐disclosure, trust, and intimacy develop stronger motivation to meet specific individuals face‐to‐face, more realistic appreciation of potential benefits, and associate with peers who similarly value authentic online connections (Angelini et al., 2024). This interpretation reframes higher engagement as adaptive rather than reckless. Adolescents who attend more meetings aren't blind to risks; rather, they may possess better reasons to meet (genuine relationships worth pursuing) or better skills to evaluate when meetings are appropriate. Future research should examine how relationship characteristics and digital skills moderate the translation of risk–benefit perceptions into behavior, potentially revealing distinct pathways to safe and beneficial face‐to‐face meetings.

Furthermore, our results suggest that adolescents' perceptions about risks, benefits, and peer involvement are interrelated. The perceived likelihood and severity of potential risks correlate positively, and both are associated with lower perceived likelihood of benefits and lower perceived peer involvement in face‐to‐face meetings. Crucially, apart from the effect of perceived likelihood of risks on perceived peer involvement, we did not observe any longitudinal within‐person effects between any of these perceptions. Thus, these between‐person correlations likely do not reflect causal effects but rather capture influences of other factors, for instance, contacts with peers who have already experienced face‐to‐face meetings. Cross‐sectional research shows that learning about these meetings from such “experienced” peers is related to lower perceived risks (Mýlek, Dedkova, & Smahel, 2023). Since face‐to‐face meetings are mostly positive experiences for adolescents (Mýlek et al., 2024; Smahel et al., 2020), adolescents who discuss these meetings with experienced peers will probably see their benefits as more likely. Knowing peers with the experience would also transfer to higher perceived peer involvement. Taken together, while perceived peer involvement does not predict participation in face‐to‐face meetings, discussing such meetings with “experienced” peers might change adolescents' perceptions about how risky, beneficial, and popular among peers these meetings are. Changes in risk and benefit perceptions could then lead to more meetings. We invite future research to examine these nuanced peer influences. While this direction seems promising, other factors, for instance, adolescents' sensation seeking or self‐efficacy, could also play a role and deserve research attention.

Lastly, we explored how age and gender relate to face‐to‐face meetings and adolescents' perceptions about them. Adolescent girls perceived the risks of these meetings to be more likely and more severe. This reflects the media discourse which portrays girls as the typical victims of “online predators” (Korkmazer et al., 2019; Mascheroni et al., 2014). Older adolescents went to more face‐to‐face meetings and perceived these meetings as more beneficial, less risky, and more common among their peers. This aligns with previous findings that the prevalence of face‐to‐face meetings increases with age (Smahel et al., 2020; Van den Heuvel et al., 2012) while perceived risks of interacting with “online strangers” and meeting them in person decrease with age (Bryce & Fraser, 2014; Mýlek, Dedkova, & Smahel, 2023). It is also in line with the idea that making new contact over the internet aligns with adolescents' developmental goals; hence, older adolescents see these meetings as more beneficial. Qualitative evidence also suggests that older adolescents feel better equipped to handle potential risks of these meetings (Bryce & Fraser, 2014), explaining the negative relationship between age and risk perception. Beyond these shifts in perception, older adolescents may also have greater opportunity to act on them. As adolescents age, they are typically granted more independent mobility and permitted to socialize without direct parental supervision, which likely facilitates arranging and attending face‐to‐face meetings with online contacts (Szwedo et al., 2017). Thus, the age‐related increase in these meetings may reflect not only more favorable risk–benefit perceptions but also greater practical opportunity to act on them.

### Limitations and future directions

Several limitations of our study need to be considered. First, when asking about the number of attended face‐to‐face meetings, we used a different timeframe in T1 (“in your life”) and in T2/T3 (“in the past 6 months”) to secure a large enough sample for a related descriptive study. To address the discrepancy in timeframes, we adjusted the T1 measure by discounting face‐to‐face meetings of adolescents who reported their last meeting had happened more than 6 months ago. The adjusted prevalence of face‐to‐face meetings in T1 was lower than in T2 and T3. This aligns with the fact that in the period before T1 data collection, contact restrictions related to the COVID‐19 pandemic were still in effect (Slabá, 2022), reducing the opportunities for in‐person meetings. Nevertheless, the adjustment of the T1 measure may have introduced some inaccuracy, and as we discussed, the pandemic context likely affected some of our findings. Additionally, our sample experienced relatively high attrition of about 33% from wave to wave. While attrition was not associated with the variables studied and is unlikely to have introduced significant bias, it likely reduced statistical power and might have affected the stability of longitudinal estimates. Thus, replications that would collect data outside the pandemic situation and ensure high participant retention are needed to corroborate our findings.

Second, to reduce the overall length of the questionnaire, we focused only on the perceived likelihood of benefits and ignored their perceived magnitude. However, our findings show that the perceived likelihood and severity of risks have different effects, and the results regarding the perceived likelihood of benefits were inconsistent with previous findings about the perceived magnitude of benefits (Baumgartner et al., 2010). Thus, we invite future studies to measure both these aspects and examine their potentially nuanced effects. Moreover, to further avoid respondent fatigue, we used brief, newly developed scales to measure risk and benefit perceptions, and single items to measure the number of face‐to‐face meetings experienced and perceived peer involvement. Although single‐item measures are adequate for capturing constructs that are unidimensional and easy to grasp (Allen et al., 2022), and our scales exhibited excellent reliability (see Table 1), future studies could improve measurement accuracy by employing more comprehensive and validated scales.

Third, our study did not focus on potential moderators. Previous research shows that face‐to‐face meetings attended with varying motives (i.e., friendly, romantic, instrumental) meaningfully differ (Mýlek, Dedkova, & Mesch, 2023), and the role of adolescents' perceptions about these different types of meetings should be further examined. Risk‐taking is also intertwined with personality characteristics, such as sensation seeking (Romer et al., 2017), that could also play a role and deserve research attention. Future studies should include relevant traits, particularly those closely related to reward‐oriented cognitions, alongside risk–benefit perceptions to empirically test whether the effects hold independently of them.

Fourth, adolescents' age may influence their perceptions of face‐to‐face meetings and their effects on behavior. Older adolescents may be more socially experienced and, accordingly, feel better equipped to handle the potential risks of these meetings (Bryce & Fraser, 2014), thereby reducing risk perceptions or attenuating their behavioral effects. On the other hand, the activities and whereabouts of younger adolescents tend to be more supervised by their parents (Szwedo et al., 2017). This may also attenuate the effects of perceptions on behavior because the decision to meet an online contact may depend in part on their level of independence or parental approval. We attempted to test the moderating role of gender, but estimation of the multigroup model failed, likely due to its high complexity. The role of age on adolescents' perceptions and behavior should be addressed in future research.

Lastly, some of the reported effects were not significant consistently across all time points. Future longitudinal research that would measure these variables across more than three timepoints is needed to confirm the robustness of our findings.

## CONCLUSIONS AND IMPLICATIONS

Meeting online contacts face‐to‐face is a common adolescent behavior, offering both developmental opportunities and potential risks. This study shows that adolescents' decisions about such meetings are shaped by their perceptions of risks and benefits, particularly the perceived severity of potential negative outcomes. Importantly, these perceptions do not develop in isolation from behavior; rather, perceptions and behavior influence each other over time, creating dynamic patterns that evolve throughout adolescence. Recognizing these bidirectional relationships challenges simplistic narratives that portray adolescents as either reckless risk‐takers who ignore dangers or as passive victims in need of protection. Instead, our findings depict adolescents as active decision‐makers who weigh potential outcomes and update their perceptions based on experience.

Our findings have practical implications for harm prevention. Effective prevention efforts must account for the complexity of adolescent decision‐making. Rather than attempting to prevent face‐to‐face meetings through fear‐based messaging, interventions should focus on helping adolescents develop skills and judgment to determine when meetings are appropriate and how to conduct them safely. This is particularly crucial given that perceptions and behavior influence each other reciprocally; once adolescents have positive experiences with face‐to‐face meetings, they may become less receptive to risk‐focused messages. Consequently, prevention efforts should engage adolescents early, before their first meeting, while providing balanced information that acknowledges both the potential benefits and the genuine, though infrequent, risks.

## AUTHOR CONTRIBUTIONS

**Vojtěch Mýlek:** Conceptualization; methodology; formal analysis; data curation; writing – original draft; writing – review and editing. **Ainize Martínez‐Soto:** Writing – original draft; writing – review and editing. **Lenka Dedkova:** Conceptualization; methodology; writing – review and editing; supervision; funding acquisition; project administration.

## CONFLICT OF INTEREST STATEMENT

The authors report there are no competing interests to declare.

## Data Availability

The preregistration, materials, analytical scripts, and data used in this study are freely available via the Open Science Framework at https://osf.io/psrfa.

## Footnotes

^1^
It is important to note that the present study does not aim to test these theoretical frameworks in their entirety and omits some of their components (e.g., attitudes, behavioral intentions, perceived behavioral control, or cues to action). Rather, we draw from these frameworks selectively, using their core logic of risk–benefit evaluation as a conceptual lens.

## APPENDIX A DETAILED RI‐CLPM RESULTS

**Table:**

Parameters	T1 → T2			T2 → T3		
	*B* (*SE*)	*p*	*β*	*B* (*SE*)	*p*	*β*
*Cross‐lagged effects*						
Benefits → FTF meetings	**0.20 (0.09)**	.**021**	.**12**	0.04 (0.08)	.595	.03
Risk likelihood → FTF meetings	−0.01 (0.06)	.818	−.01	**0.13 (0.05)**	.**007**	.**10**
Risk severity → FTF meetings	**−0.29 (0.09)**	.**001**	**−.18**	**−0.24 (0.08)**	.**003**	**−.14**
Peer involvement → FTF meetings	0.06 (0.04)	.131	.07	0.00 (0.04)	.892	.01
FTF meetings → Benefits	−0.00 (0.04)	.936	−.01	**0.17 (0.04)**	**<.001**	.**26**
FTF meetings → Risk likelihood	−0.03 (0.04)	.449	−.03	−0.05 (0.04)	.163	−.07
FTF meetings → Risk severity	−0.05 (0.03)	.116	−.08	**−0.11 (0.04)**	.**012**	**−.17**
FTF meetings → Peer involvement	−0.06 (0.06)	.304	−.05	0.12 (0.07)	.066	.11
Peer involvement → Benefits	0.02 (0.03)	.337	.05	0.01 (0.03)	.661	.03
Peer involvement → Risk likelihood	−0.01 (0.03)	.834	−.01	−0.03 (0.04)	.380	−.05
Peer involvement → Risk severity	−0.03 (0.02)	.261	−.05	0.03 (0.03)	.361	.06
Benefits → Peer involvement	0.20 (0.10)	.051	.11	−0.00 (0.12)	.965	.00
Risk likelihood → Peer involvement	**−0.16 (0.08)**	.**036**	**−.10**	0.06 (0.07)	.422	.04
Risk severity → Peer involvement	−0.02 (0.09)	.844	−.01	−0.11 (0.09)	.228	−.06
*Autoregressive effects*						
FTF meetings	0.07 (0.09)	.421	.07	**0.38 (0.08)**	**<.001**	.**38**
Benefits	0.04 (0.08)	.654	.04	0.06 (0.08)	.497	.05
Risk likelihood	0.11 (0.07)	.101	.11	**0.15 (0.06)**	.**012**	.**16**
Risk severity	0.10 (0.07)	.155	.11	0.07 (0.08)	.394	.07
Peer involvement	**0.11 (0.05)**	.**026**	.**11**	**0.13 (0.06)**	.**017**	.**13**
*Random intercept covariances*						
RI FTF meetings ↔ RI benefits	**0.06 (0.02)**	.**001**	.**48**			
RI FTF meetings ↔ RI risk likelihood	−0.02 (0.02)	.194	−.14			
RI FTF meetings ↔ RI risk severity	−0.02 (0.02)	.312	−.14			
RI FTF meetings ↔ RI peer involvement	**0.12 (0.03)**	**<.001**	.**74**			
RI benefits ↔ RI risk likelihood	**−0.08 (0.01)**	**<.001**	**−.38**			
RI benefits ↔ RI risk severity	**−0.04 (0.01)**	**<.001**	**−.25**			
RI risk likelihood ↔ RI risk severity	**0.08 (0.01)**	**<.001**	.**39**			
RI peer involvement ↔ RI benefits	**0.14 (0.02)**	**<.001**	.**58**			
RI peer involvement ↔ RI risk likelihood	**−0.06 (0.02)**	.**009**	**−.23**			
RI peer involvement ↔ RI risk severity	**−0.08 (0.02)**	**<.001**	**−.36**			
*Time‐invariant predictors*						
Gender → RI FTF meetings	0.01 (0.03)	.737	.01			
Gender → RI benefits	0.01 (0.02)	.599	.01			
Gender → RI risk likelihood	**−0.11 (0.03)**	**<.001**	**−.11**			
Gender → RI risk severity	**−0.10 (0.02)**	**<.001**	**−.12**			
Gender → RI peer involvement	0.03 (0.04)	.342	.03			
Age → RI FTF meetings	**0.05 (0.01)**	**<.001**	.**27**			
Age → RI benefits	**0.06 (0.01)**	**<.001**	.**22**			
Age → RI risk likelihood	**−0.03 (0.01)**	**<.001**	**−.11**			
Age → RI risk severity	**−0.03 (0.01)**	**<.001**	**−.13**			
Age → RI peer involvement	**0.09 (0.01)**	**<.001**	.**28**			

*Note*: Covariances, factor loadings, and residual variances are omitted. Full results are in OSF at https://osf.io/psrfa. The significance level for bold values is *p* < .05.

Abbreviation: RI, random intercept.
